# Supplementary material for: An evaluation of the introduction of telehealth for remote antenatal and postnatal contacts in Bangladesh and Lao People’s Democratic Republic during the COVID-19 pandemic
Source: PLOS Glob Public Health. 2023 May 10;3(5):e0000786. doi: 10.1371/journal.pgph.0000786 (PMC10171645; doi:10.1371/journal.pgph.0000786)
Supplement: S1 Text — (DOCX) [file pgph.0000786.s001.docx]

| **Interview Guide** | |
| --- | --- |
| **TARGETED AUDIENCE** | **KEY QUESTIONS** |
| UNFPA staff members, Maternity service advisory committee/team | 1. What planning was evident for the COVID-19 pandemic? 2. When were changes to services evident? What were they? 3. How were the UNFPA Technical Briefs and Guidance distributed in your country? 4. How were the UNFPA Technical Briefs and Guidance documents adapted for country’s response to the global pandemic? (If this occurred). 5. What other resources were useful, how did you use these? 6. Was training provided to those receiving the TBs and Guidance? 7. Due to the COVID-19 pandemic, was remote care adapted for ANC and PNC services? If so, how were they adapted, what changed, what technology/platforms were used, how did this work, and will these services be implemented in the future? (Opportunity to probe about equity). If they were not and are still underway, what do you hope for remote services? 8. What have been other innovative strategies that have come out of adapting ANC and PNC services? 9. What has been the hardest things during the COVID-19 response? 10. What has been easy? 11. What is the biggest thing you have learned about the provision of services during COVID-19 pandemic? 12. Please list two facilities, or regions that implemented remote care services (ideally an urban and a regional/rural facility) that would be interested in participating in this study. |
| Maternity care leaders, clinicians, health care workers. | 1. What were the main disruptions to the service you were providing due to the COVID-19 pandemic? 2. Did you feel adequately supported to provide women with quality antenatal/postnatal care? 3. Were you trained to adapt to the changes made to service provision? What materials/documents were used? 4. Did you provide remote care to women, was it for antenatal or postnatal care? 5. Were women responsive to remote care services? 6. What approaches are being used to overcome the disruptions to antenatal/postnatal care services in public sector health facilities? 7. What are some strategies/approaches that worked well? 8. What are some strategies/approaches that did not work as well? *(Could be phrased as: What would have made implementation better?)* 9. Do you think any of these strategies are likely to remain and change the way you provide care? 10. What support would you have liked going forward? |
| Women who accessed remote care during their pregnancy | 1. How did COVID-19 impact on your pregnancy and birth or your baby? 2. Where you able to access care in your pregnancy? If not, what were some of the barriers? 3. How was your care during your pregnancy ad after the baby was born? 4. How did you find the care provided through the phone, video, WhatsApp? 5. What were there some parts of this type of care you liked? 6. What were there some parts of this type of care you did not like? 7. What has been the hardest thing for you during this time? |
| Healthcare workers | 1. What planning was evident for the COVID-19 pandemic? 2. When were changes to services evident? What were they? 3. Which of the services were affected more drastically by the COVID-19 pandemic, antenatal or postanal care? And why? 4. Were the UNFPA Technical Briefs used in the planning and service changes? Were you trained/provided guidance as to how to use these documents effectively? 5. What was useful and what information would you liked to have seen more of? 6. What would have been good to know before the pandemic? 7. What changes should remain after the pandemic? |
